# Cracking the Code of Colorectal Cancer Screening: An Overview With a Focus on Current and Emerging Screening Methods

**DOI:** 10.7759/cureus.84724

**Published:** 2025-05-24

**Authors:** Hari Prasad Naidu Boyapati, Sidra Tahreem Hashmi Syeda, Arivudainambi Saravanan, Divya Payidiparty, Prasanna Kumar Anbazhagan

**Affiliations:** 1 Internal Medicine, People's Education Society (PES) Institute of Medical Sciences and Research, Kuppam, IND; 2 Family Medicine, Jackson Park Hospital, Chicago, USA; 3 Family Medicine, Deccan College of Medical Sciences, Hyderabad, IND; 4 Internal Medicine, Stanley Medical College, Chennai, IND; 5 Research, Shri Dharmasthala Manjunatheshwara (SDM) College of Medical Sciences and Hospital, Dharwad, IND; 6 General Surgery, Ashford and St. Peter's Hospitals NHS Foundation Trust, Chertsey, GBR

**Keywords:** colon cancer and colon polyps, colon cancer detection, colon cancer prevention, colon cancer surveillance, colon cancer treatment, colorectal cancer, colorectal cancer screening and detection, colorectal cancer surgery, metastatic colorectal cancer, rectal malignancy

## Abstract

Colorectal cancer (CRC) is a common cancer that affects the colon and rectum. It is a major cause of cancer-related mortality. A complicated interaction between environmental and genetic factors, such as age, diet, gut microbiota, obesity, chronic inflammation, and familial predisposition, appears to contribute to the pathophysiology of colorectal cancer. A multidisciplinary strategy is necessary for the effective care of CRC, including early detection through screening, surgical resection, chemotherapy, radiation therapy, and targeted medicines. The slow progression of CRC through various stages gives us, physicians, leverage and a chance to promptly and adequately screen a patient and give them a better chance at life. There are multiple clinical trials being conducted to find better and the best screening and treatment modalities. Existing and emerging screening tests can be broadly divided into blood-based, stool-based and direct imaging-based screening tests. Colonoscopy is the gold standard. However, numerous emerging screening tests are changing the face of colon cancer screening.

This review aims to highlight the background of CRC, including its etiopathogenesis, risk factors, and clinical features. It will also shed light on the existing and emerging screening techniques that will decide the future of this deadly cancer. In addition, the management section of this article will provide a summary of suggestions, including radiation and medical and surgical treatment modalities that may benefit physicians in managing this challenging condition.

We conducted a comprehensive literature search in PubMed-indexed journals using the terms "Colorectal Cancer," "Etiopathogenesis of CRC," and "Emerging screening and treatment modalities in CRC." We explored the literature on the background of CRC, its pathogenesis, the importance of screening, screening modalities and comparisons amongst each one, and treatment options for this challenging cancer. We used peer-reviewed original research articles and systematic reviews published in English, conducted from 2015 to the present time, to reflect the developments in colorectal cancer. Studies with uncertain methodological details and outcomes were excluded.

## Introduction and background

Colorectal cancer (CRC) is an invasive, malignant tumour that arises from the mucosa of the colon and rectum and is the second leading cause of cancer-related mortality worldwide [1, 2]. Although CRC was relatively uncommon in 1950, it has since become common in Western nations, where it now accounts for 10% of all cancer-related deaths [1]. Regardless of age or race, males are about 1.5 times more likely than women to acquire CRC. Intriguingly, the incidence rates of colorectal cancer (CRC) among individuals aged 20 to 49 years were 9.3 per 100,000 in 1975 and rose to 13.7 per 100,000 in 2015, representing a 47.31% change. However, the incidence of CRC has been declining among those over 50 in the US over the past few decades, while it has actually been increasing among those aged 20 to 49 [3].

A major role is believed to be played by both environmental and genetic variables in the aetiology of CRC. Nearly 75-80% of CRCs are sporadic in nature, whereas 20-25% are considered to be familial [1]. The risk of CRC is also influenced by a variety of environmental and mostly controllable lifestyle variables. Increased body weight, drinking alcohol, and smoking all raise the risk. The risk of CRC rises by 2-3% for every unit that the body mass index rises [4]. Patients who have type 2 diabetes mellitus also have a higher risk of developing CRC, and the risk of developing CRC is increased by an estimated 1.16 times for every 100 grams of red and processed meat consumed daily [5, 6].

Most CRCs originate from normal mucosa, which can lead to colorectal polyps and, in some cases, invasive CRCs. Numerous epigenetic and genetic processes that lead to the progressive silencing of tumour suppressor genes, the activation of oncogenes, and chromosomal instability cause the normal mucosa to polyp to CRC sequencing [1]. Abdominal discomfort and constipation stand out as among the more commonly presenting symptoms. However, it is unclear how to distinguish between symptoms that indicate a clear warning and symptoms that indicate a lesser risk. The relatively high specificity and sensitivity of "distended abdomen, bloating," which is mostly associated with rectal and distal colon cancer, is another significant discovery [7].

As most colon tumours arise through a complex process involving several histological, morphological, and genetic alterations that compound over time, this has made it possible to screen for and identify precancerous polyps in patients at average risk of CRC before they become cancerous. This could result in a significant reduction in the incidence of CRC [8]. The faecal immunochemical test (FIT) is the most commonly used for worldwide CRC screening. However, colonoscopy is the screening method most often utilized in the United States. In addition to FIT and colonoscopy, five blood tests are presently being investigated as prospective substitutes for CRC screening. Novel imaging tests, including the CT capsule, MR colonography, and colon capsule, are also being actively investigated [9].

Despite the diverse screening options, according to 2021 research, only 67% of patients in the USA have had their CRC screening completed and considering that 10% of all cancer-related deaths have been accounted for CRC, we need a more robust understanding of risk factors, pathogenesis and existing and emerging cancer screening modalities [1, 10]. The existing extensively used colonoscopy method is invasive, costly, and resource-intensive despite being extremely sensitive and specific for identifying CRC and removing polyps. Therefore, there is a need for multiple-modality CRC screening, which has not yet been met, to raise the existing screening rates [9].

Our Review article emphasizes the overall clinical picture of CRC and the existing CRC screening techniques, emphasizing their efficacy, dangers, advantages, and new advancements in non-invasive screening assays that are expected to transform the CRC screening landscape.

## Review

Background

Etiopathogenesis

Most CRCs are sporadic, with about 5% being caused by inherited genetic mutations, primarily Lynch syndrome (also known as hereditary nonpolyposis colorectal cancer (HNPCC)) and familial adenomatous polyposis (FAP). The development of invasive cancer typically follows a process that includes the accumulation of genetic mutations, the creation of an adenoma, and subsequent carcinogenesis. This shift from normal colon epithelium to invasive cancer takes several years [11-13]. Alternative pathways, such as those involving the BRAF gene and DNA mismatch repair gene (MMR), may be followed by some tumours [14].

The pathogenesis can be described under three distinct pathways - chromosomal instability (CIN), microsatellite instability (MSI), and CpG island methylator phenotype (CIMP) [15].

As the origin of up to 80% to 85% of all CRC cases, the CIN pathway is often referred to as the classical pathway, which will be described below [16]. It is distinguished by chromosome abnormalities that result in aneuploid tumours and loss of heterozygosity (LOH). Chromosome segregation changes, telomere malfunction, and DNA damage response are the mechanisms underlying CIN [15].

**Figure 1:**
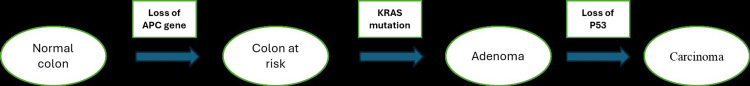
Key stages of progression in chromosomal instability (CIN) pathway. Image credit: Hari Prasad Naidu Boyapati APC - Adenomatous Polyposis Coli; KRAS - Kirsten rat sarcoma viral oncogene homolog; P53 - Tumour Protein 53

The CIN pathway is further explained in Figure 1 below.

Risk factors and clinical features

In addition to age, gender, and genetics, comorbid conditions like obesity, type 2 diabetes, and inflammatory bowel disease, as well as dietary and lifestyle factors like increased consumption of red meat and alcohol, decreased consumption of fruits and vegetables, reduced intake of calcium, decreased physical activity, and smoking, are known risk factors for CRC that may contribute to pathogenesis [17]. Reduced risk for CRC has been linked to exposure to aspirin, statins, hormone replacement treatment, and non-steroidal anti-inflammatory drugs [17]. Adenoma risk has also been linked to CRC risk factors. Still, sessile serrated lesions seem to be associated with increased age, smoking, alcohol, and decreased exposure to non-steroidal anti-inflammatory drugs [18].

Tumour location, size and presence of metastasis determine a patient's clinical presentation. Compared to right colon cancers, left colon tumours are more likely to result in partial or total intestinal blockage because the lumen of the left colon is narrower and typically contains better-formed stool because of water reabsorption in the proximal colon. Constipation, nausea, stomach distention, and abdominal discomfort are all symptoms of partial obstruction. Proximal tumours often cause occult bleeding, and patients may have iron deficiency anemia without obvious rectal bleeding. Weakness, exhaustion, dyspnea, or palpitations could be symptoms of anaemia. Cancer cachexia is a symptomatic tetrad of involuntary weight loss, anorexia, muscle weakness, and a sense of ill health that can be brought on by advanced cancer, especially when it has spread [19].

Importance of screening

Since adenoma progresses slowly to adenocarcinoma, a significant percentage of CRC cases and fatalities may be prevented. For long-term prevention against the occurrence and death of colorectal cancer, colonoscopy is still an effective screening method [20-22]. In addition to identifying malignancies in their early stages, screening techniques such as colonoscopy enable the removal of these polyps, lowering the chance that they may develop into cancer. According to systematic research referenced by the American College of Gastroenterology (ACG), screening colonoscopy was linked to a 68% decrease in CRC mortality and a pooled 69% decrease in overall CRC incidence. Additionally, the ACG points out that, among other things, CRC screening initiatives are primarily responsible for the drop in CRC incidence and mortality rates, which started in the middle of the 1980s and picked up momentum in the early 2000s [23]. Through the detection and removal of adenomas, screening programs lower the risk of colorectal cancer [20, 21, 24], and through earlier diagnosis, they improve survival and cure rates [25, 26].

This emphasizes how important screening is for early identification and prevention and, ultimately, to improve clinical outcomes.

**Figure 2:**
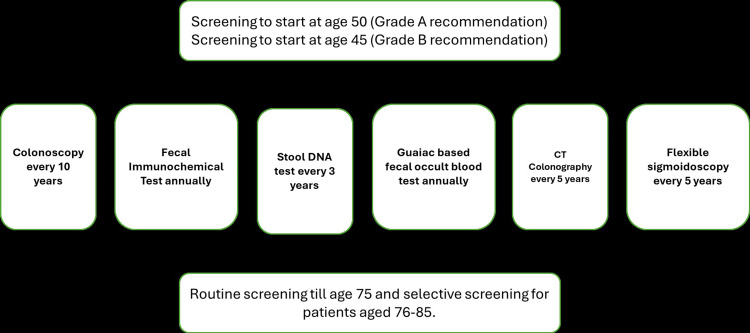
US Preventative Services Task Force (USPSTF) guidelines for colorectal cancer screening including direct and indirect modalities. Image credit - Hari Prasad Naidu Boyapati DNA - Deoxyribonucleic Acid; CT - Computed Tomography

Figure 2 includes US Preventive Services Task Force (USPSTF) guidelines [27].

Colorectal cancer screening: “Navigating the present and embracing the future”

The USPSTF now recommends seven CRC screening techniques. The multi-target stool DNA (mt-sDNA) test, faecal immunochemical test (FIT), and high-sensitivity guaiac faecal occult blood test (HSgFOBT) are among the stool-based CRC screening assays. Computed tomographic (CT) colonography, flexible sigmoidoscopy, and conventional colonoscopy are among the direct visualization screening tests. The seventh arising one is the flexible sigmoidoscopy with a FIT, which blends direct visualization with a stool-based test [28]. Examples of emerging screening methods include colon capsule endoscopy (CCE), blood-based screening assays, stool-based microbiome research, and urinary metabolomics [29]. The dynamics of colorectal cancer and ways to screen it are constantly evolving and emerging, making it a worthwhile discussion; below is a summary of all the existing and evolving screening modalities in the realm of colorectal cancer.

Existing screening modalities

Indirect Screening Tests

Sept9: A mutation in the tumour suppressor gene SEPT9, which codes for septin 9, occurs early in the CRC pathway [30]. The Food and Drug Administration (FDA) presently approves the measurement of plasma methylated septin 9 (mSEPT9), the sole blood-based test for CRC detection, for CRC screening in people who are unwilling or unable to perform the higher efficacy screening tests [31]. However, because of its insufficient sensitivity, it is not covered by the most recent USPSTF, and the US Centers for Medicare and Medicaid Services (CMS) has not authorized it for coverage [28]. The mSEPT9 assay shows non-inferior sensitivity but poorer specificity than one-time FIT for CRC and lower sensitivity for CRC and precursors in comparison to mt-sDNA testing [32].

Epi proColon: The Epi proColon test is a blood-based method for screening for CRC that looks for methylation of SEPT9 DNA in plasma. Specifically created for average-risk persons 50 years of age and older who have rejected other screening techniques recommended by the U.S. Preventive Services Task Force, it is the first and only blood test for CRC screening in the United States to receive FDA approval [33]. One nested case-control study (n=6,857) found that Epi proColon, which detects circulating methylation SEPT9DNA, had a sensitivity of 0.68 (90% CI, 0.53-0.80) and specificity of 0.79 (95% CI, 0.77-0.81) for CRC. For advanced adenomas, the corresponding sensitivity and specificity are 0.22 (95% CI, 0.18-0.24) and 0.79 (95% CI, 0.76-0.82) [34].

Guaiac-based faecal occult-blood test (gFOBT): The first stool-based laboratory test to screen for CRC was guaiac-based faecal occult blood testing. On a test card, two faeces samples are put, and hydrogen peroxide is applied. When hydrogen peroxide and heme, a catalyst, are present, guaiac, a plant resin, turns blue. Since the exam is entirely qualitative, it depends on the operator [35]. Due to a firm reliance on colonoscopy and other stool-based screening tests, there hasn't been much fresh data on gFOBT in the U.S. in recent years. According to the USPSTF-commissioned 2021 systematic study, the specificity for CRC was 0.96-0.98 (95% CI, 0.95-0.99), and the sensitivity was 0.50 to 0.75 (95% CI, 0.09-1.0) [36]. The advantages of this screening method are its affordability, accessibility, non-invasiveness, and ability to be used outside of clinical settings. To prevent abnormal results, several foods and medications must be avoided for two days before the test. These include red meat, rare meat, raw beets, carrots, cauliflower, cucumbers, grapefruit, mushrooms, broccoli, radish, horseradish, turnips, and non-steroidal anti-inflammatory drugs [37]. Smaller, pedunculated, or distal adenomas, flat or sessile adenomas, and proximal lesions are less likely to be detected by gFOBT. In contrast, it detects a higher percentage of tubule-villous and villous structures [38]. These problems have played a part in gFOBT's waning acceptance as a screening method. However, the USPSTF guidelines still include it as a primary modality every year or every three years in conjunction with sigmoidoscopy every five years. A meta-analysis conducted in 2019 on six studies spanning 15 years showed that gFOBT screening decreased CRC-related mortality (annual: RR 0.69; 95% CI, 0.56-0.86) but did not lower CRC incidence (annual: RR 0.86; 95% CI, 0.72-1.03) [39].

Faecal immunochemical test: Occult blood in faeces can also be found by faecal immunochemical testing (FIT), which agglutinates globin. When globin passes through the upper gastrointestinal tract, it is broken down, unlike heme. For lower GI bleeding, this increases FIT's specificity without allowing dietary heme sources to interfere [40]. Although many FIT products are available commercially, the OC-Sensor test (Eiken Chemical, Japan) is frequently utilized because of its comparatively high sensitivity and specificity. Sensitivity and specificity for CRC are 0.74 (95% CI, 0.64-0.83) and 0.94 (95% CI, 0.93-0.96), respectively, based on the manufacturer's suggested cut-off of 20μgHb/g faeces [36].

FIT is unaffected by a person's diet or drugs, and it only requires one stool sample per year, in contrast to gFOBT. Furthermore, since hemoglobin from foregut lesions is largely digested before it reaches the colon, it does not give an aberrant result when upper gastrointestinal bleeding occurs [41]. FIT is not without its restrictions, though. FIT should be conducted yearly for CRC screening, according to the USPSTF guidelines from 2021. This can require significant healthcare resources for patient outreach. Current data, however, suggests that FIT is superior to gFOBT. However, no interventional studies have shown a decrease in mortality yet. For non-endoscopic CRC screening, the American College of Gastroenterology (ACG) suggests FIT over gFOBT due to its anticipated advantages [42]. A 2015 cohort research (n=5,417,699) showed that biennial FIT decreased CRC mortality (RR 0.90, 95% CI, 0.84-0.95) but had no effect on CRC incidence [36].

Multi-target stool DNA testing (mt-sDNA): Over the past few years, multi-target faecal DNA testing has drawn more attention from researchers. Recently, the FDA authorized the first test that is sold commercially, called Cologuard© (Exact Sciences, Madison, WI, USA). CRC cells that have been exfoliated are shed in the stool and have a faster turnover rate than healthy cells. Cologuard© focuses on gene alterations linked to these malignant cells. These include hemoglobin, the β-actin gene (a reference for human cells), seven distinct point mutations in KRAS, and abnormally methylated BMP3 and NDRG4 [43]. The FDA approved the Cologuard test in 2014, and the US CMS has been covering it since then. The advantages of mt-sDNA include its non-invasive nature, low risk of side effects, freedom from dietary or medication limitations, and ability to be performed in non-clinical settings every three years [28, 44]. The challenges of mt-sDNA screening are its high false-positive rate in comparison to FIT and the lack of clarity surrounding the necessity of additional diagnostic testing when the result is positive, but the follow-up colonoscopy is negative, are some of its drawbacks [45, 46]. In general, mt-sDNA screening is superior to FIT in distinguishing between non-neoplastic or negative results and advanced precancerous lesions (advanced adenomas and serrated polyps ≥10mm and/or with low- or high-grade dysplasia) (p=0.004) [47].

Direct Visualization Screening Tests

Colonoscopy: In the United States, colonoscopy is the most popular method of colorectal cancer screening and the gold standard for the disease's detection. Between 30 and 75% of colorectal cancer cases are prevented with colonoscopy [48]. There is uncertainty because the process is somewhat complicated. The operator's traits and the state of the bowels determine the colonoscopy's quality [49]. In two large cohort studies, colonoscopy was linked to lower rates of CRC-related mortality (HR 0.32, 95% CI, 0.24-0.45) and CRC incidence (HR 0.53, 95% CI, 0.40-0.71) [22, 50]. Although research continuously shows that the incidence of distal colorectal cancer has decreased, the evidence supporting a decrease in the incidence of proximal colorectal cancer is less consistent [51]. However, colonoscopy reduces CRC incidence and CRC-related mortality more than flexible sigmoidoscopy does overall [51].

Flexible sigmoidoscopy: Another alternative for direct vision of the distal colon is flexible sigmoidoscopy; if polyps are seen, a referral for colonoscopy could be made. Numerous large-scale studies have compared the incidence and mortality of colorectal cancer (CRC) with repeat or one-time flexible sigmoidoscopy against no screening. Studies conducted in the UK and Italy randomly assigned 170,432 and 34,292 people, respectively, between the ages of 55 and 64, to a one-time flexible sigmoidoscopy as opposed to no screening, demonstrated a 23% and 18% decrease in CRC incidence and a 31% and 22% decrease in CRC mortality [52, 53]. About 154,887 people between the ages of 55 and 74 were randomly assigned to flexible sigmoidoscopy every three to five years as opposed to normal treatment in the USA's PLCO study, which reported a 21% and 26% decrease in CRC incidence and mortality, respectively [24]. Flexible sigmoidoscopy has several advantages over colonoscopy, such as a lower risk of bleeding and perforation, no need for sedation or anaesthesia, and a cheaper expense [51, 54]. Low adherence and the inability to evaluate the entire colon (which leaves proximal CRCs without an incidence or mortality benefit) are the main drawbacks of flexible sigmoidoscopy [51]. These drawbacks make flexible sigmoidoscopy (with or without FIT) a less popular method of screening for colorectal cancer (CRC) in the United States. Clinical settings and populations with limited access to gastroenterologists, insurance, and/or colonoscopies are the main uses for this technique [29].

Computed tomographic colonography (virtual colonoscopy): Virtual colonography visually assesses the colon and rectum for colorectal malignancies and polyps using a computed tomography (CT) scanner and computer reconstruction techniques. The recommended testing interval for CRC screening is five years, and those with abnormal results need to have a standard colonoscopy [55]. The American College of Radiology Imaging Network (ACRIN 6664) sponsored the National CT Colonography Trial (NCTC), which recruited 2,600 asymptomatic subjects for same-day optical colonoscopy and CTC. With a specificity of 85%, the sensitivity for colonoscopy-detected adenomas or colorectal cancer 10 mm or greater was 84%. For adenomas 6 mm or larger, the specificity was 86%, and the sensitivity was 70%. Lesions smaller than 6 mm were not documented, and it is unclear what the clinical relevance of these lesions is. This is one criticism of CTC [56]. The benefits of CTC include its low risk of complications, lack of procedural anaesthesia, and reduced invasiveness. Cons include radiation exposure, the need for bowel preparation, and extracolonic findings that may result in overtreatment and further testing [9]. Due to a shortage of qualified radiologists and imaging facilities to do the test, CTC's utilization is restricted [9]. Comparison of various colorectal cancer screening techniques, including their preparation needs, invasiveness, screening intervals, and diagnostic accuracy, is shown below (Table 1).

**Table 1:** Comparative diagnostic accuracy and procedural attributes of colorectal cancer screening methods. FIT - Faecal Immunochemical Test; gFOBT - Guaiac-based FOBT

Screening Method	Sensitivity	Specificity	Screening Interval	Invasiveness	Preparation Required
Colonoscopy	92–95% for cancer	~86–89%	Every 10 years	Invasive	Full bowel prep, dietary restrictions
Flexible Sigmoidoscopy	70–80% (distal lesions)	~90%	Every 5–10 years	Minimally invasive	Partial bowel prep
Faecal Immunochemical Test (FIT)	~74–88% for cancer	~94–96%	Annually	Non-invasive	None
Guaiac-based FOBT (gFOBT)	~50–75% for cancer	~96–98%	Annually	Non-invasive	Dietary restrictions before test
Stool DNA (e.g., Cologuard)	~92% for cancer	~87%	Every 3 years	Non-invasive	None
CT Colonography (Virtual)	~89–94% for large polyps	~86–93%	Every 5 years	Minimally invasive	Full bowel prep, insufflation
Capsule Endoscopy	~84% for polyps ≥6mm	~88–91%	Under investigation	Minimally invasive	Full bowel prep
Blood-based DNA (emerging)	~69–83% (varies by test)	~90–94%	Under evaluation	Non-invasive	None
Methylated Septin 9 (Epi proColon)	~68–72% for cancer	~80–90%	Every 1–3 years (pending)	Non-invasive	None

Emerging screening modalities

Indirect Screening Tests

A novel avenue for single- or multiple-cancer detection techniques is represented by blood-based cancer detection tests, commonly referred to as "liquid biopsy." Blood-based screening tests could be developed to detect CRC, which is caused by a combination of genetic and epigenetic changes in the gut mucosa [23]. As mentioned above, currently, there is only one blood-based CRC screening test that has received FDA approval - Epi proColon - and none of them have been advised for use as a first-line screening in individuals with average risk. In one nested case-control research (n=6,857), Epi proColon, which detects circulating methylation SEPT9DNA, demonstrated a sensitivity of 0.68 (90% CI, 0.53-0.80) and specificity of 0.79 (95% CI, 0.77-0.81) for CRC [34]. Plasma microRNA (miRNA) and plasma protein biomarker detection assays are the emerging methods included in blood-based screening procedures. Since miRNAs are expressed during the early stages of colorectal cancer formation, are dysregulated in precancerous and cancerous cells, and are comparatively stable in peripheral blood, they offer a special focus for CRC screening. A 2020 study of 60 patients with abnormal FIT results found that when sensitivity was set at 0.85 (no CIs were given), the specificity of a combination of miRNAs was 0.19 for high-grade adenomas and 0.26 for colorectal cancer [57]. There is also growing evidence that molecules in tumour-cell-derived exosomes, including proteins, long non-coding RNAs (lncRNAs), and microRNAs (miRNAs), are strongly associated with the initiation and progression of colorectal cancer (CRC). These molecules also reveal information about the origin of the donor cells through their content [58-60]. In addition, CRC cells generate more exosomes than non-cancer cells in vitro and in vivo, altering the immediate and distant environment and, as a result, promoting the growth and spread of tumours [61]. Compared to the screening techniques currently in use, these tests are of significant interest since they are minimally invasive, have the potential to be easily accessible, and have a high patient adherence rate [62].

For average-risk screening, stool-based microbiome tests are a new screening method that has not yet received FDA approval or been advised. People with high-grade dysplasia and colorectal cancer may have increased stool bacterial loads, which has prompted studies to find CRC bacterial indicators in stool. Like other stool-based screening methods, stool-based microbiome testing will need a follow-up colonoscopy for conclusive screening if it shows abnormalities. The fact that many of the microbiome tests available now involve genomic or metagenomic sequencing, which is more costly and time-consuming than PCR tests, presents another difficulty for this screening approach [63]. Additional investigation is required to determine the best microbiome biomarkers and biomarker combinations, as well as to assess test cost and accuracy [29].

Urine-based screening tests are another new area in CRC screening that use nuclear magnetic resonance (NMR) spectroscopy or liquid chromatography-mass spectrometry to find urine metabolites known to be linked to colorectal adenomas and colorectal cancer [64, 65]. A urine-based screening test using liquid chromatography-mass spectrometry revealed that the combination of diacetyl-spermine and kynurenine in the urine has an 80.0% specificity and a CI of 80.0% for colorectal cancer (CRC) (no CIs specified) [64]. In a study of a urine-based screening test using NMR spectroscopy, a urine metabolomic profile for adenomas was found to have an 88.9% sensitivity and a 50.2% specificity [65]. This screening method is non-invasive and allows for sample collection outside of a clinical setting [64]. However, the existing tests have subpar sensitivity and specificity, as well as their need for colonoscopy for conclusive screening in the event of an abnormal result limits them [66].

Direct Visualization Screening Tests

Colon capsule endoscopy (CCE): As a new screening method, CCE entails swallowing a wireless camera the size of a pill, which captures pictures as it passes through the digestive system [23]. While the U.S. Food and Drug Administration (FDA) has approved CCE for CRC screening in individuals with a history of incomplete colonoscopic evaluation or with a high risk of complications during colonoscopy, the USPSTF and other medical professional societies do not currently recommend it as first-line screening for average-risk individuals [23, 67]. CCE is minimally invasive, does not require sedation, insufflation, or radiation, and can be completed in a non-clinical setting [23]. CCE interpretation frequently takes longer than doing a conventional colonoscopy and writing a report, and it also calls for a practitioner with training in reading capsule endoscopy [68]. More precise and reasonably priced CCE instruments that can image a greater surface area of the colon and rectum are constantly being developed [67].

Artificial intelligence in colon cancer screening

Identifying certain colorectal lesions can be challenging, especially if they are quite small and mimic normal mucosal appearance. This makes it difficult to get a greater adenoma detection rate (ADR). Artificial intelligence (AI) is rapidly being used in detecting techniques due to the significance of enhancing ADR [69]. A meta-analysis of randomized controlled trials found that AI-assisted detection can detect 44% more adenomatous lesions than traditional colonoscopy [70]. A computer-aided detection (CADe) system called GI Genius demonstrated an ADR of 56.6% in a countrywide randomized controlled research with over 2000 patients, whereas traditional colonoscopy showed an ADR of 48.4%. The average number of adenomas discovered rose significantly from 1.21 to 1.56 (p < 0.001) [71].

Management

Local Management Including Radiation Therapy

When administered alone or in combination, neoadjuvant therapy, which includes chemotherapy and radiation, has been shown to effectively lower the tumour burden in intermediate and advanced stages of rectal cancer. There was always debate about whether the two adjuvant radiotherapies, short-course radiotherapy (RT) and long-course RT, were superior. Long-course RT is associated with higher rates of acute toxicity than short-course RT; however, there are no appreciable differences in the incidence rates of late side effects. By reducing the radiation dose, new delivery techniques, including intensity-modulated radiation therapy (IMRT), have shown promise in reducing toxicity for patients with rectal cancer and have been widely implemented in clinical practice [72].

Medical Management

Chemotherapy is used to either eradicate or prevent the growth of cancer cells. Approved cytotoxic medications for colorectal cancer prolong life and inhibit the disease's progression. Fluoropyrimidines, irinotecan, oxaliplatin, trifluridine-tipiracil, capecitabine, and 5-fluorouracil (5-FU) are among the approved drugs; they are mostly used as chemotherapeutic agents to treat colorectal cancer. At five years, the fluorouracil + L-folinic acid (FU/LV) regimen, administered daily for five days in six cycles, reduced the risk of death by 15% [73, 74].

Immunotherapy as a potentially effective therapeutic option for CRC: Since its initial successes in treating melanoma, immunotherapy has rapidly emerged as a leading therapeutic approach for several solid malignancies, including colorectal cancer [75, 76]. The specificity problem, which plagues chemotherapy, radiation, and other methods, is resolved by cancer immunotherapy. Cetuximab, panitumumab, nimotuzumab, and necitumumab are examples of antiangiogenic monoclonal antibodies that target the epidermal growth factor (EGF) receptor (EGFR). Bevacizumab and ramucirumab are antiangiogenic monoclonal antibodies targeting VEGF (vascular endothelial growth factor) and its receptor, respectively. Atezolizumab and avelumab are examples of immune checkpoint inhibitors (ICIs) that target programmed cell death ligand 1 (PD-L1). ICIs operate as immune brakes that prohibit checkpoint proteins from engaging with their companion proteins, therefore enhancing T cell effector activity [77].

Surgical Management

Depending on the location, stage, and patient-specific characteristics of the tumour, there are several surgical strategies for managing colorectal cancer. Depending on the severity of the disease, the National Comprehensive Cancer Network (NCCN) guidelines suggest several surgical approaches for rectal cancer. These include more invasive procedures like low anterior resection (LAR), proctectomy with total mesorectal excision (TME) and coloanal anastomosis, and abdominoperineal resection (APR), as well as local procedures like polypectomy, trans-anal local excision, and trans-anal endoscopic microsurgery (TEM) [78]. For patients with late-stage colorectal cancer, the American Society of Clinical Oncology (ASCO) stresses the value of a multidisciplinary team (MDT) approach. If both the primary and metastatic locations are resectable, surgery may be done with the goal of curing the condition. When available, the American Society of Gastrointestinal and Endoscopic Surgeons and the American Society of Colon and Rectal Surgeons recommend minimally invasive surgical techniques like laparoscopy.

Limitations

The effectiveness and features of both established and new colorectal cancer (CRC) screening methods are the main topics of this study; patient preferences and collaborative decision-making that affect screening uptake and adherence are not thoroughly covered. In particular, the decision between screening approaches like colonoscopy, stool-based tests (e.g., FIT, gFOBT, stool DNA), and new blood-based techniques may be greatly influenced by factors including age, comorbidities, family history, and personal beliefs. The views of patients with various comorbidities or metastatic disease, younger people with familial risk, and elderly patients were not fully investigated. Future research and clinical conversations should focus on tailored strategies that improve screening efficacy and participation by combining patient-centered considerations with clinical recommendations.

## Conclusions

Through an extensive literature review, it is evident that CRC is a scary but potentially curable malignancy when detected at the right time with the right method. This article has highlighted a full-dimensional picture of colorectal cancer, starting from its etiopathogenesis with an outline of three different pathways, including CIN, MSI and CIMP pathways, to its clinical features, risk factors and the vital significance of prompt screening. From all the reviewed studies, a conclusion can be drawn as to why CRC cancer has a substantial risk of becoming the next global health burden and how early detection and treatment can greatly lessen consequences and ease the financial and emotional strain on patients as well as healthcare systems. This emphasizes the need for more research like head-to-head studies comparing mt-sDNA and FIT, long-term outcome data on colon capsule endoscopy, or cost-effectiveness data on emerging blood tests, clinical trials, and systematic reviews about colorectal cancer, screening methods, and treatment options. While modern-age research focuses on developing the best screening methods in the market, there is a notable gap in research addressing the optimal choice amongst the screening methods available, and there is a lack of focus on the patient experience of undergoing multiple and repeated tests for accurate results. Longer-term population-based studies on screening methods and treatment options can help generations to come. This article has also reviewed most of the available screening and treatment options for physicians, along with comparisons drawn among the options which can help physicians tackle this common cancer.
